# Boolean versus ranked querying for biomedical systematic reviews

**DOI:** 10.1186/1472-6947-10-58

**Published:** 2010-10-12

**Authors:** Sarvnaz Karimi, Stefan Pohl, Falk Scholer, Lawrence Cavedon, Justin Zobel

**Affiliations:** 1 NICTA, Dept. of Computer Science and Software Engineering, The University of Melbourne, Melbourne, Victoria 3010, Australia; 2 School of Computer Science and Information Technology, RMIT University, Melbourne, Victoria 3001, Australia

## Abstract

**Background:**

The process of constructing a *systematic review*, a document that compiles the published evidence pertaining to a specified medical topic, is intensely time-consuming, often taking a team of researchers over a year, with the identification of relevant published research comprising a substantial portion of the effort. The standard paradigm for this information-seeking task is to use Boolean search; however, this leaves the user(s) the requirement of examining every returned result. Further, our experience is that effective Boolean queries for this specific task are extremely difficult to formulate and typically require multiple iterations of refinement before being finalized.

**Methods:**

We explore the effectiveness of using ranked retrieval as compared to Boolean querying for the purpose of constructing a systematic review. We conduct a series of experiments involving ranked retrieval, using queries defined methodologically, in an effort to understand the practicalities of incorporating ranked retrieval into the systematic search task.

**Results:**

Our results show that ranked retrieval by itself is not viable for this search task requiring high recall. However, we describe a refinement of the standard Boolean search process and show that ranking within a Boolean result set can improve the overall search performance by providing early indication of the quality of the results, thereby speeding up the iterative query-refinement process.

**Conclusions:**

Outcomes of experiments suggest that an interactive query-development process using a hybrid ranked and Boolean retrieval system has the potential for significant time-savings over the current search process in the systematic reviewing.

## Background

Systematic reviews of biomedical literature are a key input into *evidence-based clinical practice *whereby increasingly it is expected that medical decisions be determined by published evidence. These reviews are a summary, evaluation, and analysis of the results of published studies such as randomized controlled trials (RCTs) in relation to a highly focused medical question. Some recent example research topics, from the Cochrane collaboration, include *"Acupuncture for attention-deficit hyperactivity disorder (ADHD) in children and adolescents"*, *"Balloon angioplasty versus medical therapy for hypertensive patients with renal artery obstruction"*, and *"N-acetylcysteine for sepsis and systemic inflammatory response in adults"*.

Authoring a systematic review involves an intensive laborious process, typically involving: reading of upwards of a thousand abstracts; locating and reading of hundreds or more full papers; and assembling these into a structured document suitable for researchers and clinicians. Most such reviews are produced by a team of experts, such as senior medical researchers, and the work often takes the team a number of person-months or even -years.

A large part of the effort that goes into a systematic review is the identification of literature relevant to a given clinical question. In current practice, this involves extensive searching through bibliographic databases, such as MEDLINE or EMBASE, of medical papers - or, more often, *citations *(article abstracts plus metadata) - using complex Boolean queries. Metadata of the citations include information on each abstract, such as date of publication, language, and index terms. The current (September 2010) sizes of the major repositories are: approximately 20 million citations for MEDLINE and 20 million for EMBASE, each growing by approximately 2,000 per day; and more than 2.9 million records for PsycINFO.^1 ^In the most significant of these repositories, MEDLINE, the searchable material includes *author*, *title*, and *abstract*, and in addition most abstracts are annotated by terms from the detailed MeSH ontology of medical terms.^2^

Since exhaustive processing of such document collections is infeasible, some form of querying must be used to restrict the set of documents to be considered. The principal systems in current use are document databases supporting Boolean querying, providing operators such as wildcards and ontology-based query expansion. Reviewers use such systems to incrementally build complex queries that may involve hundreds of terms, with the aim of including the great majority of relevant documents in the answer set. Since exhaustiveness is critical, these queries may return many thousands of documents, of which perhaps one percent may ultimately meet the inclusion criteria.

The accuracy of these queries is central to the review process, and indeed these queries become part of the published review: since they precisely specify which documents were considered, a reader can use a query to assess reliability of a review, or to examine how changing the query might impact on the scope of the review.

However, these queries cannot in general provide perfect recall, and the usual tensions of information retrieval apply: we need to be able to identify all relevant documents (i.e., we require high sensitivity) without too much noise (requiring high specificity) [1,2]. While a review team might consider working through 10,000 citations, 100,000 would almost certainly be too many; and the loss of precision involved in adding new query terms may mean that it is necessary to use other mechanisms, such as browsing of specific journals, to find some of the relevant documents.

An alternative search mechanism -- one that may seem obvious to an information retrieval (IR) researcher -- is to use *ranked retrieval*. Ranked retrieval aims to order a collection of documents returned by a query such that the (most) relevant documents are towards the top of the ranking; in this case, for example, it might be satisfactory to have the majority of the relevant documents in, say, the top 1,000. However, ranking also has some potential disadvantages for the task of review generation: there is no feedback to the user as to the likely number of relevant documents; ranking cannot easily be used to combine multiple terms in a rich way; and ranked queries can be less reproducible.

### Systematic Reviews

A typical systematic review is the product of a team that has taken responsibility for analyzing the literature in a specific clinical area, usually around a highly focused issue. A team produces a stream of reviews, building on a corpus of documents collected from the relevant literature, and is also responsible for updating reviews as new literature appears. One major organization that primarily promotes the creation and publication of systematic reviews, and has developed standards to which a high quality review should adhere, is the Cochrane Collaboration.^3^

A typical systematic review usually consists of several parts: Title; Objectives; Background; Selection Criteria; Search Strategy; Data Collection and Analysis; Results; and Conclusions [3]. A review Title typically represents a specific research question; detailed research sub-questions to be answered are explained in an Objectives section, and background information in a Background section. Selection Criteria (alternatively called Inclusion Criteria) usually covers four aspects of an information need, known as *PICO*: Population, Intervention, Comparison, and Outcome. An example of a specific review title (or main research question) is "*Acupuncture for attention-deficit hyperactivity disorder (ADHD) in children and adolescents*".^4 ^This specifies "children and adolescents with ADHD" as Population, and "acupuncture" as the Intervention. Outcome (such as symptoms of ADHD) and Comparison (acupuncture versus pharmacotherapy) are listed in the selection criteria.

In constructing a systematic review, the aim is to discover all relevant documents. To make the review trustworthy, replicable, and maintainable, reviewers are required to report the inclusion criteria, exclusion criteria, and their final search strategies over the databases searched [4]. These search strategies are given in the form of Boolean queries; examples are explored later.

The literature databases used are resources such as MEDLINE, EMBASE, PsycINFO, and CENTRAL. The queries are expected to cover three aspects of the search [3]: the health condition; the intervention; and specific publication types such as Randomized Control Trials (RCTs). Selection of search terms typically involves an information specialist who iteratively modifies an initial list of selected terms, deciding how they are ordered and perhaps expanded, in consultation with the review team. Expansion usually involves either introduction of a wildcard to match variant word endings or use of the MeSH ontology (details are explained below).

### Problems with Reported Search Strategies

Despite the care taken in query formulation and reviewing of the literature, it has been observed that there are two general problems in the search process. First, the search strategies can fail to identify all relevant documents. In one study, it was found that on average 30% to 80% of published RCTs are not discovered when searching MEDLINE[5]. Another study, on a highly specific topic, showed that half of the studies were missing if only MEDLINE was searched [6]. A similar finding is also reported by McGowan and Sampson [7]. Even if MEDLINE indexed all the missing studies, the expected search strategies did not cover them all and therefore other resources - in particular EMBASE - also had to be searched. This problem is partly due to the fact that the different resources have different implementations for parsing, indexing, and searching documents, and partly because they contain different articles. A more recent study by Glanville *et al. *[8] showed that findability of a particular type of documents, randomized controlled trials, in MEDLINE has been improved since 1994. However we did not identify any study that show improved accessibility of other types of documents or databases.

In practice, reviewers use a range of strategies to find documents. In addition to searching, mechanisms include: following citations; following up entries made in registers of clinical trials; exhaustive browsing of the contents of the major journals for the area; and reading relevant conference proceedings (which might not be indexed and thus cannot be found by searching the document databases). However, the fact that these labor-intensive discovery strategies are also used does not remove the need for effective searching.

A second problem is reproducibility. Although the systematic review process ideally involves search strategies that are repeatable and can reproduce the same set of reported documents in the reviews, it has been shown that this is generally not the case [9]. For example, there are common mistakes in search strategies reported in systematic reviews that prevent them from being successfully executed via their published form. Sampson and McGowan [10] inspected 105 search strategies for MEDLINE reported in the Cochrane systematic reviews.^5 ^Not all these queries were correctly reported and only 63 out of 105 reviews could be considered for further inspection. At least one error was detected in 90% of these 63 queries, with errors being classified as: spelling errors; missed spelling variants; truncation error; logical operator error; incorrect query line references; MeSH and free-text terms in the same line; irrelevant MeSH terms; missed MeSH terms; unwarranted explosion of MeSH terms; redundancy without rationale; and search strategy not tailored for other databases. Yoshii *et al. *[11] present a complete list of studies that investigated problems in reported search strategies; percentages of unreplicable search strategies - reported for topics of reviews, databases searched, year of publication, and assessment criteria - ranged from 3.5% to 95.3%.

In the *Using Boolean Retrieval for Systematic Reviews *section, and also in our experiments reported in the results section, we investigate and present evidence for other problems with the reported search strategies, such as poor quality of reporting the Boolean queries, and also their reliance on MeSH terms which change over time and make the queries inexecutable and not repeatable.

### Search Mechanisms for Preparing Systematic Reviews

The two major paradigms available for document discovery are *Boolean search *and *ranked retrieval*. Commercial Boolean systems have been available for around forty years; ranked retrieval systems appeared rather more recently. For general search - for example, Web document search - ranking is by far the dominant mechanism, and there is a view in the IR literature that Boolean search is not as effective as ranking in *ad hoc *retrieval [12]. However, Boolean querying is currently the principal method used for searching the medical literature, and in the context of review creation both methods have strengths and weaknesses. We now explore these issues.

### Using Boolean Retrieval for Systematic Reviews

Boolean retrieval partitions a search space by identifying a subset of documents in a collection, according to the query criteria. A query is composed of a string of keywords, interspersed with Boolean operators. The simple logic means that Boolean queries are easy to process efficiently; this was of critical importance when computing power was more limited [13]. Moreover, the Boolean model is conceptually straightforward: most people have at least an intuitive understanding of sets, and for simple Boolean queries, it is usually clear to a user why a particular document does or does not match a query.

The search systems used for medical databases are not simple Boolean engines, but instead may include a variety of further operators such as word proximity, truncation or tail wildcard, and explosion or expansion. Search strategies for systematic reviews are mostly formulated for the OVID search interface, which provides a powerful interface to the MEDLINE database of medical citations. PubMed is another interface which is more general-purpose and, unlike with OVID, access is free. An example query (using OVID formulation) for the "*Acupuncture for attention-deficit hyperactivity disorder (ADHD) in children and adolescents*" systematic review mentioned earlier is as follows:

1 Attention Deficit Disorder with Hyperactivity/

2 adhd

3 addh

4 adhs

5 hyperactiv$

6 hyperkin$

7 attention deficit$

8 brain dysfunction

9 or/1-8

10 Child/

11 Adolescent/

12 child$ or boy$ or girl$ or schoolchild$ or adolescen$ or teen$ or "young person$" or "young people$" or youth$

13 or/10-12

14 acupuncture therapy/or acupuncture, ear/or electroacupuncture/

15 accupunct$

16 or/14-15

17 9 and 13 and 16

The lines in a query posed to an interface are typically numbered (1-17 in the case of the above query) to make them referable in other parts of the query. For example, line 9 refers to its preceding eight lines, combining their results in a disjunction (Boolean OR). The query contains some terms that should match exactly with any part of a document; other terms are partially represented in the query so that they match with any term in citations that have specific conditions. Examples of the first type are adhd in line 2 or brain dysfunction in line 8, where an exact match is required. The second type includes a variety of operations that indicate particular matching processes; e.g., lines 5, 6, 7, and 12 use "$" to allow matching to all the terms in the citation that start with the given string. This is similar to the use of stemming in ranked querying, where words are reduced to a root form, allowing grammatical variants of a concept to be matched. The use of stemming is normally opaque to the user; the truncation process used in Ovid is manual, since the user specifies precisely which parts of the string should be matched. In comparison to stemming, manual truncation is more powerful, but introduces additional complexity into the search process, and a greater potential for user error; e.g., if a word is truncated in the wrong place (with too few characters remaining), it will match an excessive number of documents and increase the size of the answer set dramatically.

Another type of matching is specified by the *slash *operator"**/**", which indicates search for a known MeSH heading [14], as used for example on lines 1, 10, 11, and 14 of the above query. Specifying a MeSH heading directs the system to retrieve all citations that have been manually indexed with these terms. MeSH headings are often preceded by exp (known as the explosion operator), which matches on the MeSH heading itself and all more specific terms from the MeSH hierarchy. For example, "exp acupuncture therapy" adds the following MeSH headings, and their subheading or qualifiers (not shown) to the query:

Acupuncture Analgesia

Acupuncture, Ear

Electroacupuncture

Meridians

Acupuncture Points

Moxibustion

In addition, use of the "*" operator before a MeSH heading specifies an expansion option that retrieves only articles categorized as having their main focus on the specified MeSH heading (major versus minor heading) [14].

A common problem with queries that make use of MeSH terms, and in particular the explosion operator, is that the MeSH ontology can change over time. The MeSH expansion facility in an interface such as OVID only supports the current version of the MeSH terms: if the categorization of a MeSH terms is altered, then re-running a query will retrieve a different set of results.

Not all queries are as short and simple as the one above; e.g., below is a more complex portion of a query, taken from another systematic review**^6 ^**(formulated for OVIDMEDLINE) where more specific search is targeted (in this case "vitamin B6"):

1 exp Nervous System Diseases/

2 alzheimer$

...

10 or/1-9

...

21 exp Pyridoxine/

22 (pyridoxal or pyridoxamine or pyridoxine).mp.

23 (vitamin adj1 ("B6" or "B 6" or ...)).mp.

24 or/11-23

25 10 and 24

26 limit 25 to English language

In this sub-query, the .mp. operator is used to direct the search over the whole citation (abstract, title, and subject heading words), and adj1 requires that the specified terms are directly adjacent to to one another (the numeral indicates the window size within which the terms must co-occur).

The use of fields allows a user to be even more specific. While use of .mp. specifies search over the whole citation, .tw. specifies search only in abstract and title, .ti. just in title, and .ab. just in abstract. Finally, "#" and "?" match an unspecified character inside a term (e.g. wom?n for matching both woman and women). (A full list of possible operators and features can be found in the British Medical Association MEDLINE course notes [14], and Ovid MEDLINE Database Guide by National Library of Medicine [15].)

Despite the difficulties and complexity of formulating Boolean queries, Boolean retrieval continues to be used in the process of creating systematic reviews, for a range of reasons. A key reason is reproducibility, an important factor in medical information retrieval: in principle, if the collection remains unchanged, running the same query should always return the same set of results. Another key reason is expressivity: a specific concept may be represented by a complex expression such as (clin$ adj25 trial$).ti; Boolean querying allows combinations of concepts in complex ways, and allows careful use of fields such as different components of metadata, and thus captures the semantics of the search explicitly and in ways that free-form queries cannot.

Other reasons relate to user comprehensibility. With Boolean querying, it is often obvious to the searcher why a document is included or excluded, and there is often an obvious change to the query to rectify specific inclusion or exclusion errors. The written guidelines for developing search strategies have been developed over decades, as has the experience of the creators of these strategies, creating confidence in the Boolean approach.

Finally, Boolean queries have pragmatic strengths. They should facilitate the updating of the reviews as new articles are published, and they are used to define template queries that are used to search for specific concepts. These templates can be used as sub-queries in other search strategies.

However, as discussed above, there are issues associated with the search strategies in systematic reviews that result from the Boolean querying process. The most significant of these is that it is difficult to control the number of answers. The AND operator is exclusive, so a single typographical error may remove a large portion of documents from the result set; while OR, which is inclusive, can lead to large numbers of answer documents being returned, even if these contain only a small subset of the search terms [12]. Much of the work of specifying a search strategy may not be in developing the correct semantics, but in iteratively probing answer sets and modifying the query, with the aim of minimizing loss, while trying to bring the answer set down to a manageable size.

A specific problem that this issue highlights is that it is not easy to assess the quality of an answer set. Since answer documents are unordered and there is no concept of scoring, determining whether an answer set is satisfactory or, in particular, 'final' - that is, suitable for publishing as the definitive query for a systematic review - is a difficult task.

Moreover, complex information needs are difficult to specify. Many Boolean queries used in systematic reviews are long; some examples in the Cochrane reviews are over 100 lines in length. As well as obfuscating the search logic, such queries mean that it is easy for errors to be introduced.

Finally, we note that Boolean queries such as those used for systematic reviews may not be reproducible in the longer term. One issue is that the richer operators - such as expansion, as opposed to simpler operators such as OR - may be altered in meaning, for example as new metadata fields are introduced. Another issue is that the MeSH categories are under continuous refinement.

### Using Ranked Retrieval for Systematic Reviews

The basis of ranking is that documents are scored according to evidence of relevance to an information need, typically specified by a query. There are several families of scoring function with different mathematical underpinnings, but these make use of similar information, such as: term frequency in the document; term frequency across the collection or in a background model; and (in more complex approaches) information such as local density. Further evidence can come from outside the document, such as (in the context of web querying) prior searches, link structure, or automatically inferred data such as related query terms.

Here we take a general view and describe the ranking process as assigning a score to a document that reflects the likelihood of the document being relevant to the query. Once such scores are computed, the document corpus can be ordered by decreasing score, and the *ranking *presented to the user. The user then understands that the documents of interest should tend to be towards the top of the ranking, and can proceed to inspect the documents in turn to identify which should be included in the study.

For general document search by non-expert users, there are clear advantages to using ranked retrieval, such as ease of query formulation and the fact that users typically only inspect a few documents regardless of the potential size of the answer set [16]. However, these apply best to tasks involving non-exhaustive information needs; it is unclear whether these advantages apply to the search task applicable to the construction of a systematic review.

Within the context of performing systematic reviews, we argue that an important advantage of using ranked retrieval is in the process of iterative query construction. When using ranked retrieval, the items that are most likely to be relevant are prioritized. It is relatively straightforward to see whether changes to queries have improved results, for example, by examining just the first tens of documents in an answer set, and observing whether there is sufficiently high precision at the top of the ranking. There is no equivalent feature for Boolean queries. In fact, in the Boolean case, it is extremely difficult and time-consuming to judge whether an alteration to a query leads to an improvement or harming of the answer set.

For use in systematic reviews, ranked retrieval also has some significant disadvantages. An important one is lack of reproducibility. With even a basic system, as the collection changes, so do the term statistics, and therefore the rankings: adding even a small number of new documents to a collection could potentially impact the ranking scores of individual documents. This effect could be mitigated by keeping records of the ranking functions used, in conjunction with historical version of the collection, as is done for MEDLINE.

For richer systems that make use of features such as automatic query expansion, such problems are potentially more acute; for example, the set of inferred alternatives to a query term may change significantly. Moreover, search mechanisms are frequently refined and re-tuned, and even a simple low-level change such as alteration to the parsing mechanism can lead to drastic changes in document ordering. A related problem is that it can be difficult to add mechanisms such as explicit term explosion (for example, using the MeSH ontology) to a scoring function in a consistent way, and it is possible that the semantics of explosion will change over time. Relatedly, ranking algorithms can appear complicated and opaque to the searcher: it can be difficult for a user to determine or understand why the system has chosen to return documents in a particular order, especially in the presence of expansion or explosion.

A more significant issue is that, for a rich query, the result set is in effect an ordering of the entire collection. In principle this should lead to a reduction in workload: instead of having a large unordered set of candidates to work through, under ranking the most useful items should tend to appear early in the list. However, as we explore in our experiments described below, to some extent a user may continue to gain information by perusing the answer list for even tens of thousands of documents, and indeed is unlikely to achieve high recall without doing so. There is no boundary that defines some documents as excluded, and no clear way to determine when to stop inspecting an answer list. Indeed, as our experiments show, 'reasonable' mechanisms for deciding when to stop examining results (such as a continuous run of a thousand irrelevant documents) lead to ranked retrieval having rather worse performance than Boolean searching.

Our challenge, then, is to more precisely understand the limitations of both ranked and Boolean search, and to use this understanding to propose mechanisms that reduce the labor of constructing systematic reviews without losing relevant documents. In the following sections we describe a detailed investigation using a sample query set to quantify the limitations of both approaches to search, within the context of the task of constructing systematic reviews.

## Methods

In the Results and Discussion section, we report on a number of experiments using a ranked retrieval search engine, and a number of search strategies, and compare these to baseline Boolean search results obtained using the OVID interface to MEDLINE.

To perform ranked retrieval experiments, we used an open-source search engine called Zettair^7 ^which has most popular ranking functions implemented. We used the Okapi BM25 similarity algorithm [17] (default settings) as our ranking function, for all experiments. Armstrong *et al. *[18] showed that BM25 outperforms most other ranking algorithms for ad hoc retrieval. Okapi is a probabilistic function that ranks the matching documents to a given query based on their relevance.

### Data and Measurement

For our experiments, we used a publicly available dataset^8 ^of 15 systematic reviews, created by the US Agency for Healthcare Research and Quality (AHRQ) on drug-related topics, assembled by Cohen *et al. *[19] -- we call this the *Drug *dataset. Another set of 12 systematic reviews were compiled from publicly available reviews from AHRQ^9 ^to further validate our observations -- we call this the *Misc *dataset. Our criteria in choosing reviews were: clear listing of included and excluded studies; and clearly specified OVID MEDLINE search strategies. The specific included and excluded studies, as listed in the final reports, are taken to be the gold standard of items that a search strategy should retrieve. In this analysis, we are not concerned with the potential existence of other relevant documents in the collection that were not included in the systematic reviews.

In our experiments, we search for articles in the National Library of Medicine (NLM) MEDLINE bibliographic collection, which contains the *citations *(abstract plus metadata) of medical journal papers. The copy of MEDLINE used was updated in late November 2008, and contained 17,132,315 unique citations. We refer to each *citation *as a *document*.

A number of different evaluation metrics have been proposed for the evaluation of search performance. These are generally based on two underlying characteristics of a search result set: *precision*, defined as the number of relevant documents that the search system has retrieved, divided by the total number of documents that were retrieved; and *recall*, the number of relevant documents that were retrieved, divided by the total number of relevant documents that are available in the collection. Recall, by definition, assumes full knowledge of all relevant items in a collection. While systematic reviews strive for completeness, it is extremely unlikely that all possible relevant documents that have been written on a topic will to be retrieved. However, as explained, we take the list of those papers that are included in a systematic review as a gold standard.^10 ^The term "recall" is therefore potentially misleading: it is possible to find 100% of all papers that were included in a review; but this is not the same as claiming that every single relevant document has been identified. In this text anywhere we mention recall, we mean fraction of included documents.

For comparability, precision and recall are reported at different cutoff levels *N *as P@N and R@N; the thresholds are chosen to match the typical size of Boolean query output sets when searching for documents to include in a systematic review (typically 1,000 and 10,000 when retrieving from the whole MEDLINE collection).

*Rank-Biased Precision (RBP) *is a precision-focused metric that can be adjusted with a *persistence parameter p*, to reflect the expected patience of users as they work their way down a ranked list of search results [20]. We use a value of *p *= 0.99 in our experiments, modeling a patient user who is willing to review an extensive answer set; this reflects the current behavior of users engaged in systematic review construction, who work their way through large sets of documents returned by Boolean queries.

As explained above, the creation of a systematic review can be viewed as consisting of three stages:

1. First, an initial search strategy is employed to retrieve a large pool of potentially relevant citations from databases such as MEDLINE;

2. Second, human experts scan the set of citations (consisting of abstracts plus metadata), and based on these identify a smaller set of candidate documents that meet the specified inclusion criteria;

3. Third, human experts examine the full text of the smaller set of documents, to identify those studies that are then considered for inclusion in the final systematic review document.

Based on this process, we can identify three levels of relevance judgments, based on information reported in each review:

1. *Tier 0 *is the set of documents that are retrieved by original search strategies (the Boolean queries) reported in the reviews, using the OVID MEDLINE interface;

2. *Tier 1 *is the set of documents that the reviewing experts identified as relevant by screening title and abstract;

3. *Tier 2 *represents included studies as reported in the final review.

Note that these levels only represent sets of documents and not the actual process behind finding those documents. Using these three levels is informative because it allows us to compare retrieval performance at different stages of the review process. In particular, Tier 0 compares the output of a ranked system with the original Boolean search strategy, while Tier 2 shows effectiveness in terms of finding the final included papers. For the latter, a good retrieval system should obtain close to 100% recall. The numbers of papers available at each tier for each review in our test collections are summarized in Table 1.

**Table 1:** Description of test datasets sourced from AHRQ.

	**Drug Dataset**			**Misc Dataset**		
		
**Review**	**Tier 0**	**Tier 1**	**Tier 2**	**Tier 0**	**Tier 1**	**Tier 2**
						
1	2,544	183	41	616	38	12
2	851	84	20	1,733	273	92
3	310	92	16	32,308	421	198
4	1,120	363	146	4,949	413	83
5	2,072	302	42	10	508	104
6	1,218	279	100	505	130	34
7	368	80	80	1,261	1,103	440
8	393	88	41	14,935	796	65
9	1,915	48	15	10	329	21
10	503	139	136	243	535	121
11	1,333	238	51	1,235	2,329	365
12	1,643	34	9	682	158	77
13	3,465	173	85			
14	671	218	24			
15	327	78	40			
						
Avg.	1248.5	159.3	56.4	4537.2	604.2	138.2

The number of relevant documents per query in each tier is listed. For Drug Dataset Tier 0 represents original sets of documents found by Boolean queries. Tier 0 statistics for Misc Dataset are based on Boolean queries executed by us on OVID, and does not represent the original set that the reviewers have seen.

We use the Zettair search engine, using the Okapi BM25 similarity algorithm for ranking, to retrieve the 10,000 top ranked documents for each query made for the *Drug *set of 15 reviews. Tier 0 results allow us to compare the different ranked queries with the original Boolean queries (that is, searching from the full MEDLINE collection to retrieve the initial large pool of candidate documents).

### Ranked Querying

To examine whether ranked querying could be a plausible alternative to the current Boolean retrieval paradigm for the review process, we first need to identify how to formulate an effective ranked query. Although ranked queries are conceptually easier to formulate than Boolean queries (for example, since no use of special Boolean operators is required), it is not clear how very complex information needs should be represented. We consider a number of different approaches for the formulation of ranked queries for searching in the context of systematic reviews. We then investigate two common techniques that may enhance retrieval effectiveness: incorporating metadata, and query expansion.

#### Formulating Ranked Queries

While published reviews include the Boolean query used to obtain Tier 0 documents, we need to define a systematic way to construct ranked-search queries appropriate for each review in our test set. As explained in the background section, a systematic review starts with a highly focused clinical question that typically appears as the review Title. The Background section of a review provides the detailed research questions, and agreed definitions of each of the possible PICO elements. A list of inclusion and exclusion criteria accompanies each review. Since this information is proposed before the reviewing process commences, we would expect it to be a good candidate for formulating ranked queries.

For our experiments, we constructed three sets of ranked queries, incorporating increasing amounts of information:

1. Title only (T);

2. Title and background information, in the form of detailed research questions (TR); and

3. Title, research questions, plus inclusion criteria (TRC).

**Figure 1:**
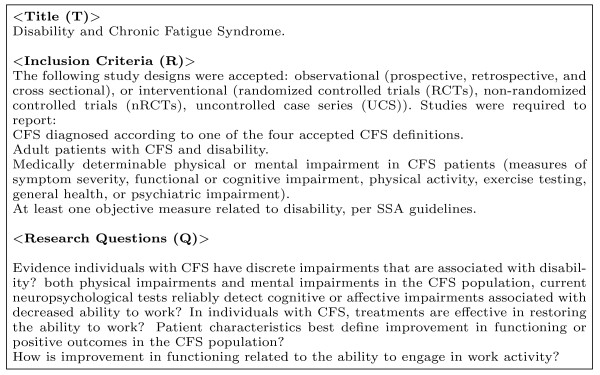
**A sample ranked query (TRC) based on an AHRQ systematic review (Drug Dataset)**. Note the headings only show where the query words are sourced and do not represent any structure to the query. The query is treated as a "bag-of-words", that is, the entire text is entered as a query without imposing any structure or word order.

A sample of a TRC query is given in Figure 1.

A second approach to formulating ranked queries is to simply co-opt the original Boolean queries as specified in the review; this was done by extracting all index terms and removing the Boolean operators (B).

## Results and Discussions

In this section, we report on a number of experiments that compare the performance of Boolean search and ranked retrieval. Our investigations include: various ways of constructing search queries from reported reviews; the utility of exploiting metadata in MEDLINE records; and using MeSH terms for query expansion. We also report on performance of a possible *stopping criterion *for determining when to stop examining ranked documents.

### Experiment 1: Effectiveness of Ranked Query Schemes

The effectiveness of all four types of queries (T, TR, TRC, and B) are shown in Table 2. Low recall values -- for all three types of ranked queries based on structured information needs -- demonstrate the low degree of overlap between the outputs of the Boolean and ranked-retrieval systems, at any given rank. The best performance is obtained by TRC queries, with recall score of 15.5% when considering the top 1,000 documents, and 46.1% when considering the top 10,000 documents, as shown in Table 2.

**Table 2:** Effectiveness of different ranked queries in each tier, searching from the full MEDLINE collection.

**Index**	**Target**	**Query**	**RBP99**	**P@1,000**	**R@1,000**	**P@10,000**	**R@10,000**
							
MEDLINE	Tier 0	T	0.043	0.038	0.042	0.021	0.202
		TR	0.092	0.079	0.088	0.040	0.380
		TRC	0.166	0.124	0.155	0.046	0.461
		B	0.150	0.118	0.143	0.044	0.408
							
MEDLINE	Tier 1	T	0.010	0.009	0.064	0.004	0.296
		TR	0.024	0.021	0.161	0.008	0.575
		TRC	0.067	0.044	0.327	0.011	0.709
		B	0.053	0.040	0.284	0.010	0.676
							
MEDLINE	Tier 2	T	0.003	0.003	0.082	0.001	0.283
		TR	0.008	0.008	0.195	0.003	0.601
		TRC	0.036	0.019	0.409	0.004	0.784
		B	0.019	0.014	0.341	0.004	0.718

Index indicates the source of documents, while Target indicates the relevant documents that the evaluation is based on. For example, Tier 1 in the Target column indicates that we judge our system based on the documents that are chosen for full-text screening.

In general, using all three types of structured queries, precision in top rank positions is low (**<**5%) for both Tier 1 and Tier 2 searches. Overall, it can be seen that TRC queries, which incorporate more information to try to identify relevant documents, perform better than the T and TR queries in terms of precision and recall (i.e., in place of recall). We therefore use only the results of TRC queries in the the following experiments.

Ranked queries that are derived by simply taking all terms from the original Boolean queries, B, show performance that is lower than the structured queries, TRC. This is surprising, especially when considering the recall measure: the ranked version of the original queries should include all documents defined by the original Boolean set (since the entire ranking should include all documents that contain at least one query term, equivalent to using the "OR" Boolean operator between all terms). This motivated us to investigate the robustness of the original search strategies in Experiment 5 explained below.

Considering retrieval from MEDLINE to Tier 2 (that is, identifying the relatively small set of documents that are actually included in the final systematic review) shows similar trends: TRC queries outperform other variants, and show a relatively high proportion of identified documents (recall of 40.9% for the top 1,000 documents, and 78.4% for top 10,000 documents).

Examining effectiveness for searching between the different tiers provides information about how effective ranking is at supporting different stages of the systematic review creation process. We simulate the last two steps of this process by creating an indexed collection consisting of only Tier 0 documents, and then querying for Tier 1 and Tier 2 documents; and then indexing Tier 1 documents and querying for Tier 2 documents. The performance for the different ranking techniques is shown in Table 3. Given the smaller number of documents in Tiers 1 and 2 (refer to Table 1), for this set of experiments we report appropriately lower cut-off values for precision and recall.

**Table 3:** Effectiveness of different ranked queries on two sub-sets of MEDLINE corresponding to different stages of the systematic review process.

**Index**	**Target**	**Query**	**RBP99**	**P@500**	**R@500**	**P@10,000**	**R@10,000**
							
Tier 0	Tier 1	T	0.215	0.149	0.565	0.104	0.684
		TR	0.261	0.195	0.713	0.136	0.885
		TRC	0.301	0.209	0.747	0.139	0.895
							
Tier 0	Tier 2	T	0.090	0.066	0.553	0.043	0.690
		TR	0.121	0.082	0.748	0.052	0.926
		TRC	0.148	0.090	0.819	0.053	0.944
							
				P@50	R@50	P@150	R@150
							
Tier 1	Tier 3	T	0.249	0.381	0.440	0.265	0.705
		TR	0.280	0.411	0.495	0.297	0.847
		TRC	0.290	0.445	0.518	0.310	0.872

Index indicates the source of documents, while Target indicates the relevant documents that the evaluation is based on. Given Tier 1 was smaller set of documents than Tier 0, we used smaller thresholds for the evaluation.

Similar to the retrieval experiments from full MEDLINE, TRC queries perform better than T and TR queries, but the difference is not as significant. The cut-off of top 500 returned documents represents approximately 50% of Tier 0 documents; therefore, around 82% of the included studies are ranked in the top 50% of the documents when the output of the original Boolean queries are ranked (Tier 0 indexed, Tier 2 target). Clearly, our experiments retrieving Tier 2 from the Tier 1 result set do not completely match the manual process of reviewing, in which full-text documents are assessed against the inclusion and exclusion criterion and PICO specifications (our ranking algorithms continue to use only citations - abstract plus metadata - for each tier). Consideration of the full text, as well as more advanced algorithms that process the text based on the specified inclusion or exclusion criteria in TRC queries, is expected to improve the process further.

### Experiment 2: Effectiveness of Exploiting Metadata

The Boolean query-based search strategies that are currently used to retrieve an initial (i.e., Tier 0) set of candidate documents for inclusion in a systematic review are generally formed by a search expert, who has specialized domain knowledge. These queries therefore commonly include criteria about the metadata that is associated with entries in the underlying collection (e.g. MEDLINE citations). Examples of metadata include features such as: limiting results to documents that were written or published in certain time-periods; limiting results to studies on humans; or limiting results to specific languages (see *Using Boolean Retrieval for Systematic Reviews *Section for examples).

We explore two approaches to ranked searching with explicit control over metadata. In the first approach, MEDLINE metadata fields are indexed separately (Method A) by the Zettair search engine; in this method, terms in the ranked query can be explicitly marked to indicate whether they should match the metadata or the text of the citation abstract. The second approach involves indexing metadata separately, but considers it to be part of the freely searchable abstract text content (Method B). This has the advantage of allowing higher weighting to be given to specific metadata such as *inclusion criteria*, but also enables the matching of general query terms that may occur in the metadata (for example, in index terms) if they are mentioned in the abstract. This method is therefore more tolerant to possible mistagging of metadata in the query.

The results for retrieval from MEDLINE are shown in Table 4. We hand-tagged the TRC queries based on the same tagging scheme carried out on the MEDLINE collection. Method A performed better than method B, but did not show large improvements compared to the baseline shown in Table 2. We therefore conclude that separate indexing for metadata is not helpful in retrieval for the systematic review search task. These results are in line with other studies that show little or no benefit in using MEDLINE metadata for biomedical information retrieval [21,22].

**Table 4:** Retrieval effectiveness when metadata is tagged explicitly for ranked querying.

**Target**	**Method**	**RBP99**	**P@1,000**	**R@1,000**	**P@10,000**	**R@10,000**
						
Tier 0	A	0.184^† ^	0.135	0.159	0.051^† ^	0.483
	B	0.115^‡^	0.100^‡^	0.101^† ^	0.044	0.402^† ^
						
Tier 1	A	0.079^† ^	0.049^† ^	0.343	0.011	0.752^† ^
	B	0.036^‡^	0.029^‡^	0.203^‡^	0.009^† ^	0.625^‡^
						
Tier 2	A	0.041^† ^	0.022^† ^	0.442	0.005	0.856^‡^
	B	0.014^‡^	0.012^‡^	0.259^† ^	0.004^† ^	0.689^† ^

In Method A, metadata is indexed separately; Method B searches metadata separately, but also includes it as part of the abstract text. All results are shown for searching from MEDLINE to the Target level indicated. †, ‡ indicate paired t-test with 95 and 98 percent confidence levels respectively (over baseline of TRC queries).

### Experiment 3: Effectiveness of Query Expansion Using MeSH

Query expansion can improve search performance by helping to overcome keyword mismatches. We investigate the use of automatic query expansion based on MeSH for the identification of relevant documents for systematic reviews.^11 ^This is similar to the expansion techniques used in Boolean retrieval, as described in the background section of this paper.

We used the US National Library of Medicine (NLM)'s Medical Subject Headings (MeSH) ontology to expand the baseline ranked queries. We tested three approaches for query expansion based on different MeSH components:

1. Using MeSH qualifiers (sub-headings) only;

2. Combining qualifiers and concept terms; and

3. Using qualifiers, concept terms, abbreviations and supplementary terms from the MeSH hierarchy.

We used the Genia part-of-speech (POS) tagger [23] to identify noun phrases in the queries. These identified phrases were then searched for in the MeSH thesaurus, using exact match of the phrase. All identified terms from a matching MeSH thesaurus entry were added to the initial query, which was then re-submitted to the search engine.^12^

Retrieval results are presented in Table 5, showing retrieval from the MEDLINE collection to different Target tiers. Compared to the results in Table 2, using MeSH query expansion in this way does not lead to any improvement in retrieval effectiveness.

**Table 5:** Effectiveness of query expansion using MeSH.

**Target**	**Src**	**RBP99**	**P@1,000**	**R@1,000**	**P@10,000**	**R@10,000**
						
Tier 0	1	0.162	0.124	0.157	0.043	0.449
	2	0.173	0.131	0.160	0.045	0.436
	3	0.179	0.130	0.162^† ^	0.045	0.440
						
Tier 1	1	0.066	0.043	0.320	0.010	0.673
	2	0.069	0.046	0.335	0.010	0.700
	3	0.074	0.045	0.335	0.011	0.682
						
Tier 2	1	0.035	0.019	0.405	0.004	0.769
	2	0.036	0.020	0.421	0.004	0.790
	3	0.038	0.020	0.416	0.004	0.758

Retrieval is from the full MEDLINE collection, to the Target level indicated. The TRC queries are expanded, using three sources (Src): 1, qualifiers (sub-headings); 2, qualifiers and concept terms; and 3, qualifiers, concept terms, abbreviations, and supplementary terms. POS tagging is applied to detect phrases in the queries. †, ‡ indicate paired t-test with 95 and 98 percent confidence levels respectively (over baseline of TRC queries).

### Experiment 4: Defining a Stopping Criteria

A key disadvantage of ranked queries is that their result set can be very large. Any document that contains at least a single query term may obtain a similarity score assigned to it; in the extreme case, where a long query contains many common terms, the result set may include the entire collection of documents.

In the effectiveness analysis of the previous section, we reported precision and recall at cut-off levels chosen to approximate the total number of included documents that are available at each Tier; this simulates the case where the user arbitrarily stops viewing result documents after having seen a certain number of them. However, when a new search is being carried out, this number is not known in advance. This raises the question of how many documents in a ranked results list should actually be inspected for the review process.

In systematic reviewing, reviewers are patient users that inspect all the results returned by Boolean queries with precision as low as a fraction of one percent. For example, assuming that the final included documents are spread uniformly across the result set of the original Boolean search strategy, the precision for query 12 of the Drug dataset is 0.5%.

The required patience of a reviewer can be analyzed in a ranked system by considering a *tolerance threshold *[20]. We define *tolerance *to be the number of non-relevant items that a user has seen since seeing the previous relevant item; the tolerance threshold is then the number of (consecutive) non-relevant items a user is willing to observe before discontinuing this search.^13 ^If the number of consecutive non-relevant items exceeds the tolerance threshold, the user assumes that the system has no more relevant items to show, and stops. Conversely, if a relevant item is seen before the threshold is reached, the tolerance counter is re-set.

**Figure 2:**
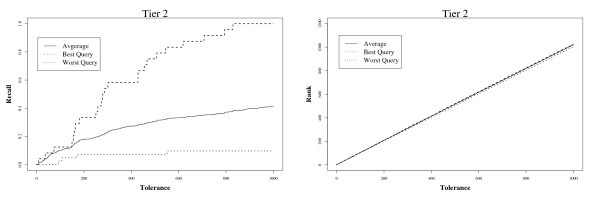
**Tolerance versus recall (left), and rank (right).** Lines show the relationship for the best (query 14), worst (query 1), and average over 15 TRC queries, when retrieving from the full MEDLINE collection. Evaluations are based on final included studies (Tier 2).

Tolerance is plotted against recall in the top half of Figure 2. The left-hand side shows retrieval from MEDLINE to Tier 1, and the right-hand side shows retrieval to Tier 2. The best and worst performing queries, and the average over the 15 Drug queries are shown when TRC ranked queries are used. For the best case (query 14), if the tolerance threshold is 830 or higher, 100% of the included Tier 2 studies are found. For the worst case (query 1), even with a threshold of 1,000 the density of included documents in the ranking is so low that the user would find only around 10% of the total before giving up.

The rank position of the irrelevant documents which cause a tolerance threshold to be violated is shown in the lower part of Figure 2.

**Figure 3:**
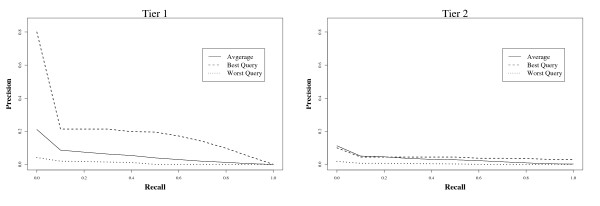
**Average recall versus precision using TRC ranked queries on MEDLINE**.

Figure 3 shows a plot of precision against recall. Even for effective queries, it is apparent that precision falls rapidly after the first few included documents are found by the ranked queries. On average, however, precision is very low even at low levels of recall.

Overall, these results suggest that even very patient users are unlikely to find a large proportion of the included documents within a reasonably bounded portion of the ranked answer set. It therefore seems that a simple ranked retrieval system is not an adequate replacement for current Boolean search systems: despite the intuition that ranking should reduce effort by placing relevant items near the start of the list, precision is so low overall that reviewers would need to work through infeasibly large numbers of non-relevant documents to be sure of having located all documents that should be included in a review. In this search scenario, a key strength of the Boolean approach is therefore that it is able to provide a restricted list of documents to assess. In the next experiment we investigate a possible way to bound the result set by combining ranked-retrieval and Boolean search approaches.

### Experiment 5: Combining Boolean and Ranked Retrieval

In the previous section, it was shown that a key problem with the use of ranked retrieval was the increased size of the result set that needs to be examined to ensure that all included documents are found. A potential solution to this problem is to combine Boolean and ranked querying, for example, by first running a Boolean search strategy, and then applying ranking to this bounded result set.

As a first step, we attempted to replicate the search strategies as included in the systematic reviews that are used for analysis. However, when these strategies are re-run, the result sets often do not manage to retrieve the final included Tier 2 documents that are listed in the systematic review. One explanation is that the reviewing team often finds some of the relevant documents through other methods, such as checking the citations of the articles known to be relevant.

Even without replacing the Boolean paradigm, we believe there are improvements that can be made to the search *process *by incorporating aspects of the ranked-retrieval search process. In particular, one could make use of the simple query form, and the use of a ranking schema to quickly determine potential of result lists in providing relevant information. The complexity of the Boolean queries often leads to their execution failure after some minor changes in the medical databases (for example, updates of MeSH headings). Such complexity, which is introduced primarily to narrow the search space to make it manageable for the reviewers, can mistakenly cause removal of some interesting studies.

In this section, we explore refinements of the standard Boolean search paradigm. We first investigate the effect of the query complexity in our case study and examine possible ways to eliminate it. Based on the refined initial Boolean searches, we then investigate the application of ranked retrieval to the original bounded candidate document set, and its utility in identifying the efficacy of a Boolean search.

### Experiment 6: Unnecessary Complexity in Search Strategies

There is much debate on how to form Boolean queries to strike an appropriate balance between specificity and sensitivity [1,2] output of a search strategy must be judged by human assessors with a finite amount of financial and time resources, it is vital that the size of an answer set size does not exceed some feasible limit. Boolean operators play an important role in both locating the relevant studies and restricting the size of the result set. The impact of the Boolean operators on recall is not quantified in any study, with some only suggesting very broad advice on how to approach the search problem with predefined patterns. For example, McGowan and Sampson [7] state that, if one is searching for RCTs, the search should be tailored to match only with subject headings; if not, it will end up finding papers *about *randomized controlled trials. Such a strict searching paradigm is very reliant on the metadata and how it is stored for each article. We investigate the impact of imposing many restrictions on the search strategy; our experiments indicate that adding too many parameters can cause the elimination of many relevant documents from the set of results.

**Figure 4:**
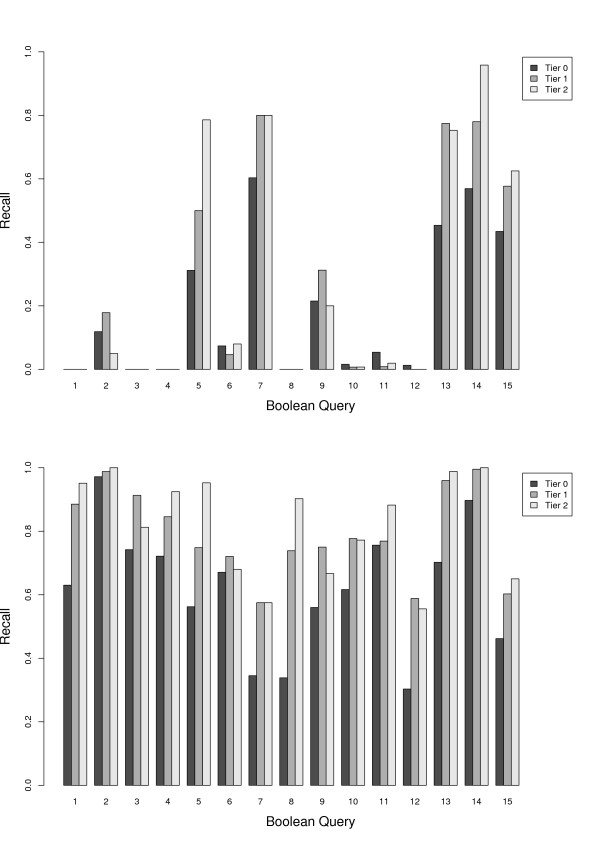
**Recall of Boolean queries after re-running on Ovid MEDLINE (1950-Nov week 3, 2008)**. Average recall for (a) original queries (upper) are 0.27 and 0.29 for Tier 1 and Tier 2, respectively, and for (b) simplified queries (lower) are 0.79 and 0.82.

We first re-ran the Ovid MEDLINE Boolean queries of each review using the Ovid interface to see whether we could retrieve the same set of documents reported in the review. Figure 4(a) shows per-query recall for the *original *Boolean queries reported in the reviews. Overall, we had trouble replicating the search results: few included documents could be found, with average recall in Tier 1 equal to 27%. Five queries out of 15 returned no relevant documents in all three tiers and only query 14 had 100% recall in Tier 2. We investigated the hypothesis that the presence of restrictive operators causes the low recall in these queries. If this is the case, then we would expect their removal to improve recall. Thus, we modified the queries to first generalize some of the keyword matchings, and then removed the limits on *publication type*, *dates *, and so on. Table 6 summarizes the most common Boolean operators in search strategies, their functionality, and our method of generalizing them. We refer to the modified queries as *simplified *in our experiments.

**Table 6:** Common Boolean queries operators and their replacements used for simplifying - finding supersets of - the queries.

**operator**	**meaning**	**action**
		
/or .sh.	MeSH heading	remove or replace with .mp.
$	truncate (similar to stemming)	unchanged
limit to *x*	limit the search to some *x *conditions (e.g language or publication type)	remove line
.ed.	entry date	remove line
adj *n*	two specific words separated by maximum *n *words	make them separate terms connected by or
.mp.	match term with subheadings, title, and abstract	unchanged
.ti.	match term with title words	replace with .mp.
.tw.	match term with title or abstract terms	replace with .mp.
.fs.	match term with floating (two-character) subheadings	replace with .mp.
and	conjunction	replace with or (not always)
or	disjunction	unchanged
exp	explode using MeSH subheadings	unchanged

As an example, the following original lines from a query:

21 exp pyridoxine**/**

22 pyridoxal.ti.

23 (vitamin adj1 ("B6" or "B 6")).mp.

24 or**/**21 -- 23

25 random:.sh,pt.

26 limit 25 to English language,

are simplified to the following:

21 exp pyridoxine**/**

22 pyridoxal.mp.

23 (vitamin or "B6" or "B 6").mp.

24 or**/**21 -- 23.

We executed the simplified queries using the Ovid interface on MEDLINE (1950-Week 3, 2008). Figure 4(b) shows per-query recall for the Boolean queries simplified with rules listed in Table 6. From the results it can be seen that recall for all the queries has increased (the average recall in Tier 1 increased from 27% to 79%). As a trade off, we had larger result set for these queries. The changes in the number of retrieved documents per query is listed in Table 7. Retrieved items in the results set of the queries were not unique: some duplicates were introduced by multiple queries per review, and some were the result of redundancy in the database output. Table 7 shows both the total and the unique number of documents returned.

**Table 7:** The output size of the original and simplified Boolean queries in the drug collection after re-running on Ovid MEDLINE (1950 - Week 3, Nov 2008).

			**Simplified**		
			
	**Original**		**Ovid**		**Lucene**
			
**Query**	**all**	**unique**	**all**	**unique**	
					
1	2,153	1,986	29,074	15,027	7,654
2	1,378	1,347	120,420	111,653	1,596
3	101	101	1,857	1,799	1,568
4	994	897	12,857	11,924	81,371
5	1,526	1,343	63,494	53,319	8,093
6	276	276	159,772	153,121	36,686
7	1,200	1,117	109,584	104,743	308,329
8	309	230	106,154	81,018	64,704
9	1,464	1,464	41,544	35,618	17,686
10	64	64	2,260	2,205	243,733
11	930	540	6,420	6,172	23,896
12	140	140	3,215	3,058	1,599
13	5,578	5,378	21,332	20,588	13,491
14	1,969	1,782	39,446	36,487	24,230
15	997	997	9,859	9,568	5,189

Number of unique documents returned and total number of retrieved documents are shown per query.

Although a Boolean system does not provide any special ranking and only provides some sorting capabilities based on specific fields (e.g., *date*)^14^, we tried to estimate the effort of finding the relevant documents in the generated output. Table 8 shows the effectiveness of both the original and simplified Boolean queries for the 15 queries run using the Ovid interface on MEDLINE. The simplified queries are markedly higher in terms of included documents retrieved, with Tier 2 recall of 82%, compared to only 28% for the original queries. Precision, in the context of Boolean retrieval, can be used to provide an overall ratio of included to not-included documents; to avoid any bias that the Ovid default sorting may introduce, the outputs of both result sets were ordered randomly. Although the simplified queries generated much larger result set sizes, they were comparable to the original queries in terms of precision (Tier 2).

**Table 8:** Effectiveness of Boolean retrieval approaches: un-ordered documents using original and simplified Boolean queries (Ovid).

	**Level**	**Precision**	**recall**
			
Boolean	Tier 1	0.0282	0.2656
(Orig)	Tier 2	0.0121	0.2853
			
Boolean	Tier 1	0.0130	0.7904
(Simp)	Tier 2	0.0058	0.8209

(a) original search strategies (b) simplified search strategies

A further complication to reported search strategies is added by the presence of the *explode *operator. Using this operator, the Ovid interface allows expansion of the query terms using the MeSH subject headings: this is enforced when a known MeSH term is prefixed by *exp*. The functionality of this operator, however, is not clearly defined in terms of which levels of terms in the ontology are added to the query. We adapted the simplified queries to Boolean queries that the Lucene search engine^15 ^could execute. However, due to the ambiguity of the expansion operator, we decided to remove these operators from the queries. The effectiveness of the system is shown in Table 9. Recall and precision show improvements over the Ovid output, both for the original and simplified queries. We draw two conclusions from this experiment: first, *exp *has no effect in increasing the effectiveness compared to the original and simplified Boolean queries; second, comparing the **trc **ranked queries with ordered Boolean results, ranked queries are more effective in terms of precision, and are competitive in terms of recall.

**Table 9:** Comparison of effectiveness of different retrieval approaches: Boolean only systems (Original and Simplified), ranked only retrieval (TRC queries), ordered simplified Boolean queries (Lucene), combined approach (simplified Boolean queries on Ovid and ranked querying using Zettair).

**Method**	**Level**	**RBP99**	**P@1,000**	**R@1,000**	**P@10,000**	**R@10,000**
						
Boolean	Tier 1	0.026	0.026	0.179	0.004	0.266
(Orig)	Tier 2	0.009	0.010	0.202	0.001	0.285
						
Boolean	Tier 1	0.015	0.014	0.103	0.007	0.426
(Simp)	Tier 2	0.008	0.006	0.115	0.003	0.414
						
Ranked	Tier 1	0.067	0.044	0.327	0.011	0.709
(TRC)	Tier 2	0.036	0.019	0.409	0.004	0.784
						
Boolean	Tier 1	0.023	0.023	0.172	0.013	0.528
(Lucene)	Tier 2	0.008	0.007	0.197	0.004	0.577
						
Combined	Tier 1	0.084	0.057	0.415	0.011	0.677
	Tier 2	0.048	0.028	0.532	0.004	0.765

### Experiment 7: Ranking the Boolean Output

We investigated a combined method that benefits from the main property of Boolean retrieval in restricting the search space to a subset of the MEDLINE collection, but offers a ranked output. Similar approaches have previously been studied for general information retrieval (IR) systems, for example, *extended Boolean IR *by Salton *et al. *[24]. The combined Boolean/ranked-retrieval system first produces an unordered list of documents using simplified Boolean queries on the Ovid interface. It therefore guarantees relatively high recall values. Then, using the TRC ranked queries we try to order the subset of retrieved documents and push the most relevant documents to the top of the list. The performance of this approach is shown in Table 9. The results show that the combined system successfully moves the scattered relevant documents towards the top 50% of the list (the top 50% contained 75.5% of the total 82.1% of the Tier 2 relevant documents initially found using the Ovid interface over MEDLINE).

**Figure 5:**
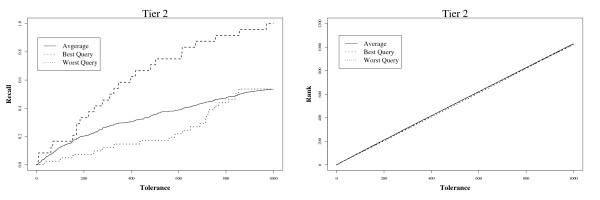
**Tolerance versus recall (left), and rank (right) for two queries (best and worst), and average over 15 TRC queries.** Retrieval was over subsets of MEDLINE determined by the simplified Boolean queries. Evaluations are based on final included studies (Tier 2).

**Figure 6:**
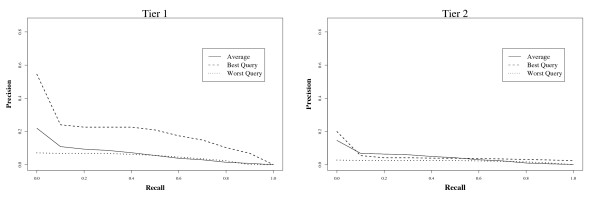
Average recall versus precision using TRC ranked queries on the subset of MEDLINE retrieved by their corresponding Boolean queries.

Figure 5 shows recall versus tolerance level for the combined system. The relationship between tolerance and rank is shown in Figure 5. Precision versus recall is shown in Figure 6. Query 1 -- which was the worst performing using the pure ranked-retrieval system (TRC queries) and one of the worst for the original Boolean queries -- had a boost in recall (9.7% to 53.6%, Tier 2 results). Still the system does not guarantee 100% recall, and precision is low (**<**8% for Tier 1 results).

### Experiment 8: Effect of Miscellaneous Queries

The experiments reported in the previous sections all used the Drug dataset which contained 15 queries with similar topics. We repeated our experiments using a set of queries that do not share a theme--i.e., using queries derived from the *Misc *Dataset (Table 1).

**Figure 7:**
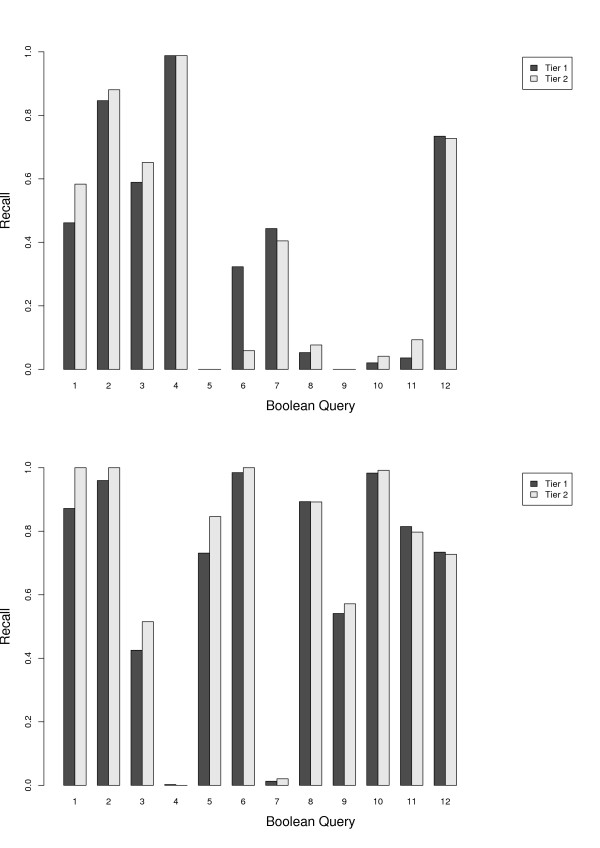
Recall of the Boolean queries in the Misc dataset after running the original (upper) and simplified (lower) Boolean queries on OVID MEDLINE.

Many of the search strategies reported in these reviews were not replicable in their entirety (mostly due to inaccurate reporting of the search strategies, such as non-existent MeSH headings). The simplified queries were construed based on the original (sometimes erroneous) queries. Figure 7 shows the recall performance for the original and simplified queries in the *Misc *dataset. Except for two queries (Query 5, and Query 10), all other queries benefited from simplification. For the majority of queries, the increase in performance was significant. The size of the records retrieved for each query is shown in Table 10 -- the result set size increased for all except two queries (Queries 5, and 9).

**Table 10:** The number of retrieved documents using OVID for the original and simplified boolean queries of Miscellaneous dataset.

	**Tier 0**	
	
**Review**	**Orig**	**Simp**
		
1	628	14,244
2	1,691	96,797
3	31,552	22,213
4	4,813	1,881
5	10	78,536
6	507	91,572
7	1,174	40,930
8	14,952	7,938
9	11	74,200
10	243	278,832
11	1,235	496,656
12	662	983

The results of retrieval using the Boolean, ranked, and combined systems are shown in Table 11. Ranked retrieval using the **trc **queries shows improved effectiveness compared to all other methods that rely on the original Boolean queries.

**Table 11:** Effectiveness of different retrieval methods, including using ranked only and hybrid ranked and Boolean, on the Misc dataset. Lucene results are over 12 queries.

**Method**	**Level**	**RBP99**	**P@1,000**	**R@1,000**	**P@10,000**	**R@10,000**
						
Boolean	Tier 1	0.065	0.070	0.151	0.010	0.250
(Orig)	Tier 2	0.032	0.027	0.262	0.003	0.350
						
Boolean	Tier 1	0.015	0.060	0.200	0.011	0.500
(Simp)	Tier 2	0.005	0.020	0.310	0.004	0.560
						
Ranked	Tier 1	0.060	0.072	0.185	0.023	0.516
(TRC)	Tier 2	0.036	0.030	0.306	0.007	0.645
						
Boolean	Tier 1	0.070	0.040	0.116	0.030	0.319
(Lucene)	Tier 2	0.029	0.015	0.150	0.007	0.404
						
Combined	Tier 1	0.055	0.065	0.200	0.028	0.485
	Tier 2	0.020	0.026	0.300	0.006	0.553

## Conclusions

Evidence-based medicine makes use of clinical evidence from reliable scientific sources, such as systematic reviews of high-quality randomized controlled trials, in clinical practice on patients. A systematic review is a highly systematized literature review on a focused clinical question that identifies, appraises, selects and synthesizes all the relevant high quality research evidence; this is a highly time-consuming process. It is to be hoped that improving search mechanisms for systematic reviewing can therefore enhance the quality of the evidence it provides, and to reduce the time taken to perform the process.

In this paper, we have explored how ranked retrieval may be used to support the search problems encountered when creating systematic reviews. Our results show that the performance of bag-of-words queries (i.e., the simple queries used in ranked-retrieval) can be improved significantly by adding research questions and inclusion criteria. We have also investigated the impact of using tagged metadata terms in order to improve search performance. Our results suggest that where such terms are available, they should be matched with both their specific MEDLINE citation fields, as well as the full text. Query expansion based on the MeSH thesaurus was also investigated, but the variants we evaluated had no observed beneficial effect on retrieval performance. Moreover, our results have demonstrated that the impact of different ranked querying techniques is affected by the stage of the review process (or tier) at which the searching takes place, as well as the type of systematic review report from which the query is derived. Our experiments also demonstrate that ranked queries offer comparable or better recall than the reported search strategies when re-run subsequent to the report generation.

A key drawback of ranked retrieval is that the size of the result set is likely to be much larger than that returned as the result of a Boolean search strategy. This is problematic, since the number of documents that a team of reviewers can examine is limited. Investigating a tolerance threshold - the number of non-useful documents that a reviewer sees before deciding to stop - indicated that the precision of ranked retrieval systems must be higher in order to offer a useful alternative to the current search process used in the creation of systematic reviews.

We therefore investigated a hybrid approach, where a Boolean search strategy is used to fetch an initial pool of candidate documents, and ranking is then applied to order the result set. Our results show that applying ranking to this bounded set can successfully pull relevant documents towards the top of the list, therefore potentially reducing the workload of reviewers by providing an early indication of result set quality.

Current work involves codifying an alternative process to systematic search: i.e., enabling interactive development of effective boolean queries via the techniques described in Experiment 5. We believe this to be a key approach to facilitating higher-performance search for the purpose of compiling systematic reviews, potentially leading to significant time-savings in the search phase of this task.

## Competing interests

The authors declare that they have no competing interests.

## Authors' contributions

SK and FS collected the datasets. SK and SP ran experiments. All authors participated in development of the method, experiment design, and writing. All authors read and approved the final manuscript.

## Appendix

^1^Review authors also make use of resources such as databases of registered studies, where text searching is relatively of less importance; we do not consider such resources in this paper.

^2^MeSH (the taxonomy of Medical Subject Headings) consists of a hierarchical set of around 25,000 descriptors, used to support the indexing of and searching for biomedical and health-related information (http://www.nlm.nih.gov/pubs/factsheets/mesh.html, accessed 15 July 2009).

^3^The reviews, published in a highly structured form, are available at http://www.cochrane.org/reviews/ (accessed 16 Feb

2010).

^4^http://www.mrw.interscience.wiley.com/cochrane/clsysrev/articles/CD007839/frame.html (accessed 16 Feb 2010)

^5^http://www.cochrane.org/reviews/ (accessed 16 Feb 2010)

^6^B Vitamins and Berries and Age-Related Neurodegenerative Disorders: http://www.ncbi.nlm.nih.gov/bookshelf/br.fcgi?book=erta134 (accessed 27 Sep 2010)

^7^http://www.seg.rmit.edu.au/zettair (accessed 16 Feb 2010)

^8^http://davinci.ohsu.edu/~cohenaa/systematic-drug-class-review-data.html. (accessed 16 Feb 2010)

^9 ^Our assembled collection can be accessed in: http://www.csse.unimelb.edu.au/~skarimi/SystematicReview.html

^10^Note that Sampson *et al. *[25] use the term *relative recall *to refer to a measure of evaluation that follows our (mentioned) methodology of making a gold standard which uses only MEDLINE citations .

^11^Researchers have also considered query expansion using the Unified Medical Language System (UMLS) thesaurus [26,27].

^12^We also investigated using the same process with a direct match between individual terms and MeSH terms -- that is, without initial POS tagging of the query. The results were similar to using the POS tagger, and are not shown here.

^13^In the context of web-search, reaching the threshold would lead to the user submitting a new query.

^14^This is only in cases where the result set is not too large.

^15^http://lucene.apache.org/ (accessed 16 Feb 2010)

## Pre-publication history

The pre-publication history for this paper can be accessed here:

http://www.biomedcentral.com/1472-6947/10/58/prepub

## References

[B1] Haynes B McKibbon KA Wilczynski NL Walter SD Werre SR Optimal search strategies for retrieving scientifically strong studies of treatment from MEDLINE: analytical survey British Medical Journal 2005 330 7501 1179 1182 10.1136/bmj.38446.498542.8F 15894554 PMC558012

[B2] Zhang L Ajiferuke I Sampson M Optimizing search strategies to identify randomized con-trolled trials in MEDLINE BMC Medical Research Methodology 2006 6 23 10.1186/1471-2288-6-23 PMC1488863 16684359

[B3] The Cochrane Collaboration Cochrane Handbook for Systematic Reviews of Interventions, Version 5.0.0 2008 http://www.cochrane.org/resources/handbook/

[B4] Klassen TP Jadad AR Moher D Guides for reading and interpreting systematic reviews: I. Getting started Archives of pediatrics and adolescent medicine 1998 152 7 700 704 9667544

[B5] Dickersin K Scherer R Lefebvre C Systematic Reviews: Identifying relevant studies for systematic reviews British Medical Journal 1994 309 6964 1286 1291 7718048 10.1136/bmj.309.6964.1286 PMC2541778

[B6] Avenell A Handoll H Grant A Lessons for search strategies from a systematic review, in The Cochrane Library, of nutritional supplementation trials in patients after hip fracture American Journal of Clinical Nutrition 2001 73 3 505 510 11237924 10.1093/ajcn/73.3.505

[B7] McGowan J Sampson M Systematic reviews need systematic searchers Journal of the Medical Library Association 2005 93 74 80 15685278 PMC545125

[B8] Glanville JM Lefebvre C Miles JN Camosso-Stefinovic J How to identify randomized controlled trials in MEDLINE: ten years on Journal of the Medical Library Association 2006 94 2 130 136 16636704 PMC1435857

[B9] Brazier H Poorly executed and inadequately documented? An analysis of the literature searches on which systematic reviews are based Second Symposium on Systematic Reviews: Beyond the Basics 1999 Oxford, United Kingdom 5 7

[B10] Sampson M McGowan J Errors on search strategies were identified by type and frequency Journal of Clinical Epidemiology 2006 59 10 1057 1063 10.1016/j.jclinepi.2006.01.007 16980145

[B11] Yoshii A Plaut DA McGraw KA Anderson MJ Wellik KE Analysis of the reporting of search strategies in Cochrane systematic reviews Journal of the Medical Library Association 2009 97 21 29 10.3163/1536-5050.97.1.004 19158999 PMC2605027

[B12] Cooper WS Getting beyond Boole Information Processing and Management 1988 24 3 243 248 10.1016/0306-4573(88)90091-X

[B13] Witten IH Moffat A Bell TC Managing Gigabytes: Compressing and Indexing Documents and Images 1999 2 Morgan Kaufmann Publishers

[B14] BMA British Medical Association (BMA) Library - MEDLINE Plus, Basic Course - Notes for OvidSP http://www.bma.org.uk/images/medlinesp2010_tcm41-199937.pdf (accessed 27 September 2010) 2010

[B15] NLM Ovid MEDLINE Database Guide http://www.ovid.com/site/products/ovidguide/medline.htm (accessed 24 September 2010)

[B16] Jansen BJ Spink A Saracevic T Real life, real users, and real needs: a study and analysis of user queries on the web Information Processesing Management 2000 36 2 207 227 10.1016/S0306-4573(99)00056-4

[B17] Walker S Robertson SE Boughanem M Jones GJF Jones KS Okapi at TREC-6 Automatic ad hoc, VLC, routing, filtering and QSDR TREC 1997 125 136

[B18] Armstrong T Moffat A Webber W Zobel J Has adhoc retrieval improved since 1994? Proceedings of the 32nd international ACM SIGIR Conference on Research and Development in Information Retrieval, Boston, MA 2009 692 693 full_text

[B19] Cohen A Hersh W Peterson K Yen PY Reducing workload in systematic review preparation using automated citation classification Journal of the American Medical Informatics Association 2006 13 2 206 219 10.1197/jamia.M1929 16357352 PMC1447545

[B20] Moffat A Zobel J Rank-biased precision for measurement of retrieval effectiveness ACM Transactions on Information Systems 2009 27 1 27 10.1145/1416950.1416952

[B21] Abdou S Savoy J Searching in MEDLINE: Query expansion and manual indexing evaluation Information Processesing and Management 2008 44 2 781 789 10.1016/j.ipm.2007.03.013

[B22] Lu Z Kim W Wilbur J Evaluation of query expansion using MeSH in PubMed Information Retrieval 2009 12 69 80 10.1007/s10791-008-9074-8 19774223 PMC2747526

[B23] Tsuruoka Y Tsujii J Bidirectional inference with the easiest-first strategy for tagging sequence data Proceedings of the conference on Human Language Technology and Empirical Methods in Natural Language Processing Vancouver, Canada 2005 467 474 full_text

[B24] Salton G Fox EA Wu H Extended Boolean information retrieval Communications of the ACM 1983 26 11 1022 1036 10.1145/182.358466

[B25] Sampson M Zhang L Morrison A Barrowman N Clifford T Platt R Klassen T Moher D An alter-native to the hand searching gold standard: validating methodological search filters using relative recall BMC Medical Research Methodology 2006 6 33 10.1186/1471-2288-6-33 PMC1557524 16848895

[B26] Hersh W Bhupatiraju RT TREC 2003 Genomics Track Overview The Twelfth Text REtrieval Conference, Gaithersburg, MD 2003 14 23

[B27] Zhu W Xu H Hu X Song IY Allen R Using UMLS-based re-weighting terms as a query expansion strategy IEEE International Conference on Granular Computing, Atlanta 2006 217 222

